# Toothed whales independently evolve specialized suction feeding without converging on skull shape

**DOI:** 10.7717/peerj.21119

**Published:** 2026-04-17

**Authors:** Rebecca J. Strauch, Alexander J. Werth, Carlos Mauricio Peredo, Mark D. Uhen

**Affiliations:** 1 Department of Atmospheric, Oceanic, and Earth Sciences, George Mason University, Fairfax, VA, United States of America; 2 Department of Biology, Hampden-Sydney College, Hampden-Sydney, VA, United States of America; 3 Department of Institutional Analytics and Research, Western Governors University, Salt Lake City, UT, United States of America

**Keywords:** Aquatic feeding, Morphology, Feeding ecology, Cetacean, Parallel evolution

## Abstract

Specialized suction feeders independently evolved in several odontocete (toothed whale) lineages, with paradigmatic examples in sperm whales and beaked whales. Although little is known about feeding behavior in most cetacean species, many studies have identified morphological correlates of feeding ecology, such as skull shape. Here, we develop a suction specialization index (SSI) to quantify the extent of suction specialization in 68 extant odontocete taxa. This metric incorporates 10 morphological characters historically associated with suction feeding, including skull and hyoid osteology, dentition, and soft tissue structures. Our analyses show that both SSI and individual morphological characters reliably distinguish suction-feeding odontocetes from other taxa. Reduced dentition, non-occluding teeth/jaws, specialized palate, and weak jaw adductors were the strongest predictors. The blunt, wide rostrum was the weakest predictor. The distribution of SSI in the morphospace demonstrates that lineages of suction-feeding odontocetes specialize without converging on skull shape. Our results suggest that quantifying specialization across a suite of morphological predictors can provide a robust framework for reconstructing feeding ecology in extinct taxa. Our findings also highlight the interplay of convergence and constraint in vertebrate feeding systems, ultimately contributing to a broader understanding of how specializing on feeding behavior (e.g., suction) manifests across many different forms.

## Introduction

The pervasiveness of suction feeding among aquatic vertebrates exemplifies widespread functional convergence across disparate body plans (Wainwright et al., 2015). Suction feeding is well-documented in numerous lineages of fishes (Motta & Wilga, 2001; Lauder, 1982; Bemis & Lauder, 1986) and amphibians (Deban & Olson, 2002; Heiss et al., 2013), as well as several lineages of secondarily aquatic tetrapods (Marshall, Kovacs & Lydersen, 2008; Werth, 2000b; Enstipp et al., 2018; Summers et al., 1998). At its core, suction feeding involves capturing and transporting prey using intraorally generated subambient pressures (Wainwright et al., 2015). Suction is a solution for feeding in a dense, incompressive fluid environment, where bow waves created by forward locomotion would otherwise push prey out of reach (Ferry-Graham, Wainwright & Lauder, 2003; Werth & Marshall, 2023).

Most secondarily aquatic mammals employ suction to some extent in the feeding process (*e.g.*, transporting grasped prey to the back of the oral cavity) (Werth, 2000a; Hocking et al., 2017), with many converging on feeding strategies that use suction for prey capture (Marshall, Kovacs & Lydersen, 2008; Werth, 2000b; Werth & Marshall, 2023; Kastelein, Muller & Terlouw, 1994; Kane & Marshall, 2009; Bloodworth & Marshall, 2005). Specialized suction feeding has repeatedly evolved in odontocetes (toothed whales), with notable examples in extant sperm whales and beaked whales (Werth, 2004; Heyning & Mead, 1996). Although little is known about feeding behavior in the vast majority of cetacean taxa, suction performance has been observed or experimentally demonstrated in several species (Werth, 2000b; Kane & Marshall, 2009; Bloodworth & Marshall, 2005; Kastelein et al., 1997). Other studies have identified a number of morphological traits associated with suction feeding (Reidenberg & Laitman, 1994; Werth, 2006b; Werth, 2006a; Werth, 2007; Johnston & Berta, 2011). Here, we build on this work by testing whether suction feeding can be inferred from morphology alone.

Reliable predictions of suction feeding from morphology are critical for interpreting the ecology of species for which feeding behavior cannot be directly observed. Many suction specialists feed at depth (Tyack et al., 2006; Watwood et al., 2006; Arranz et al., 2019), which poses severe limitations for studying feeding behavior in the field. Testing performance in captive animals *via* controlled experiments is extremely difficult and not possible for the majority of taxa. For fossil cetaceans, inferences of suction feeding rely solely on morphological data (Fordyce, 2002; Boessenecker et al., 2017; Lambert et al., 2017; Peredo et al., 2018).

**Figure 1:**
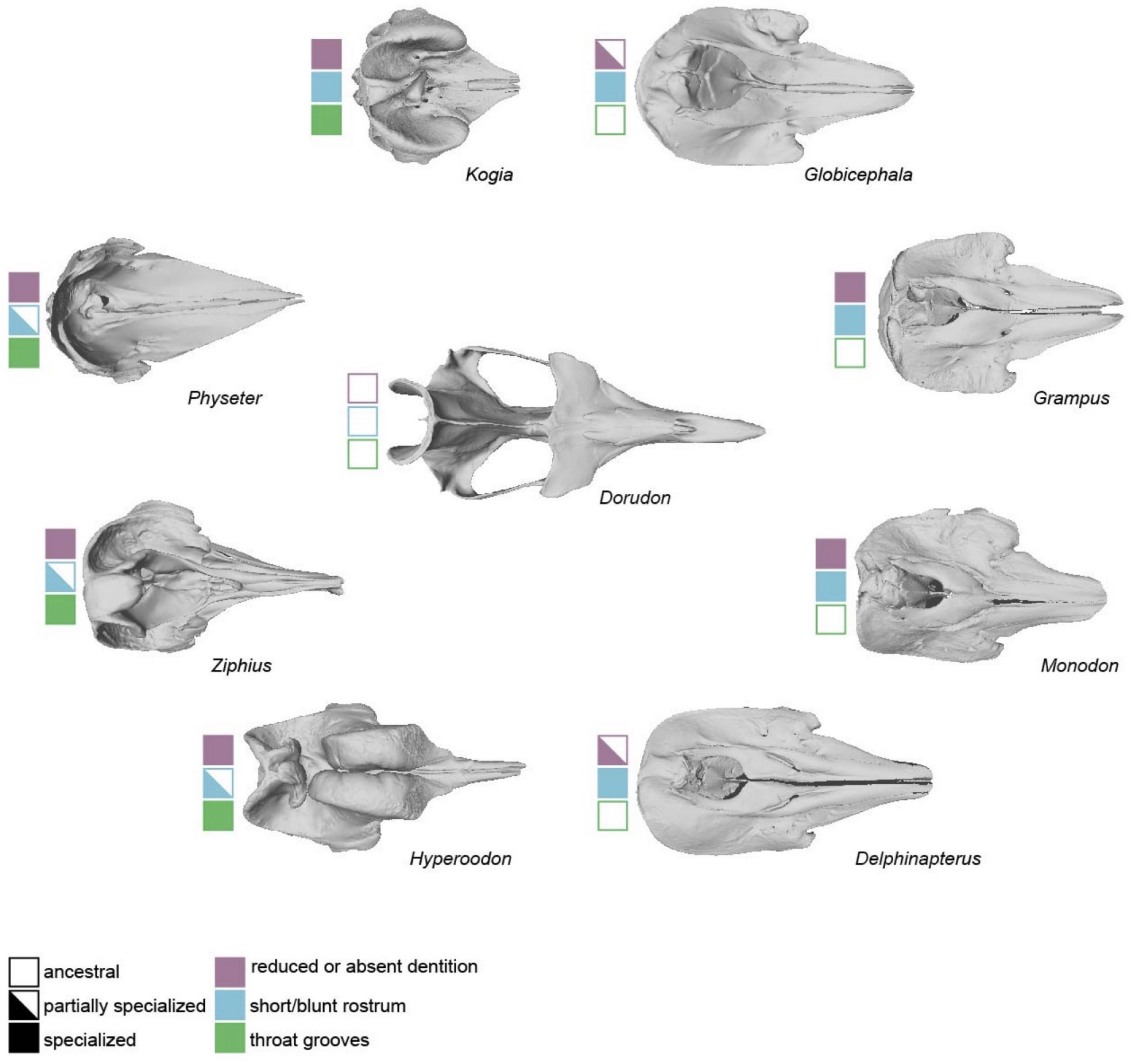
Cranial diversity of specialized suction-feeding odontocetes. Three-dimensional skull models representing cranial diversity across suction-feeding odontocetes. Squares indicate the expression of three suction-related morphological characters: reduced or absent dentition, wide/blunt rostrum, and throat grooves. The skull of a stem cetacean (*Dorudon atrox*) represents an ancestral condition that is not morphologically specialized for suction feeding. Scans of *Hyperoodon ampullatus* (NHMUK ZD 1992.42), *Physeter macrocephalus* (NHMUK ZD 2007.100), and *Ziphius cavirostris* (NHMUK 1915.7.20.1) were acquired through Sketchfab courtesy of Richard Sabin. Scans of *Delphinapterus leucas* (USNM VZ 22207), *Globicephala macrorhynchus* (NHMUK ZD 1912.10.27), *Grampus griseus* (USNM VZ 24224), *Kogia breviceps* (USNM VZ 22015), and *Monodon monoceros* (USNM VZ 267959) were acquired through Phenome10k. *Dorudon atrox* (UMMP VP 118139) was provided courtesy of the University of Michigan Museum of Paleontology.

Capture suction feeding evolved in parallel in several odontocete lineages, including sperm whales (Physeteridae and Kogiidae) (Bloodworth & Marshall, 2005; Werth, 2004), beaked whales (Ziphiidae) (Heyning & Mead, 1996), belugas and narwhals (Monodontidae) (Werth, 2000a; Kane & Marshall, 2009), pilot whales (*Globicephala*) (Werth, 2000b; Kane & Marshall, 2009), Risso’s dolphin *(Grampus griseus*) (Werth, 2000a; Werth, 2006a), and harbor porpoise (*Phocoena phocoena*) (Kastelein, Muller & Terlouw, 1994). Although these taxa converge on prey capture method, they nevertheless span a range of skull morphologies (Fig. 1). This disparity has implications for identifying specialized suction feeders from a suite of morphological predictors.

Cranial and mandibular shape have been linked to feeding ecology in numerous vertebrate groups (Liem, 1993; Pierce, Angielczyk & Rayfield, 2009; Wroe & Milne, 2007; Emerson, 1985), including whales (McCurry et al., 2017b; Galatius et al., 2020; Coombs et al., 2024). However, diverse skull morphologies among “obligate” suction feeders suggest that specialized suction feeding does not constrain skull shape. Here, we develop a suction specialization index (SSI) to quantify the extent of suction specialization in 68 extant odontocete taxa. We then examine the distribution of SSI in the morphospace to test whether suction specialization coincides with convergence on skull shape.

## Materials & Methods

### Suction-related character scores

We sourced morphological data from the primary literature for 68 extant odontocete species, spanning all 10 extant families (Table S1). We supplemented this dataset with A.J.W.’s observations from necropsies and museum specimens, also published in the literature cited here (Werth, 2000b; Werth, 2000a; Bloodworth & Marshall, 2005; Werth, 2004; Reidenberg & Laitman, 1994; Werth, 2006b; Werth, 2006a; Werth, 2007; Johnston & Berta, 2011; Ito et al., 2002; Bloodworth & Marshall, 2007; Werth & Beatty, 2023; Loch, Fordyce & Werth, 2023). We scored species for 10 morphological character traits historically associated with suction feeding: blunt, wide rostrum, reduced dentition, dental wear, non-occluding teeth/jaws, robust tongue/hyoid, specialized palate, weak jaw adductors, long mandibular symphysis, round mouth, and throat grooves. Although there are likely other suction-related traits, we selected these characters because previous studies have identified strong correlations with suction. For each trait, taxa were assigned a score ranging from 0 to 3, where scorings reflect the degree to which the trait is expressed (0 = never present, 1 = weakly present, 2 = somewhat present, 3 = strongly present). For throat grooves, taxa were assigned either 0 (never present) or 3 (strongly present).

### Quantifying morphological specialization

We quantified the extent of suction specialization in odontocetes using a new index developed here: the suction specialization index (SSI). The SSI was calculated for each taxon as the mean score across all 10 suction-related characters (CharMean), with an alternative metric based on the sum of character scores (CharSum) used for comparison. To evaluate the index’s utility for fossil taxa, we also calculated an adjusted SSI. This metric simulates the conditions observed in fossil taxa by excluding the two soft-tissue characters (round mouth and throat grooves), relying on the remaining eight osteological and dentition characters instead. We therefore compared two versions of the SSI: an index representing the average score across all 10 morphological characters, and an adjusted index representing the average score across the eight osteological and dentition characters. Both SSI values were calculated by dividing the sum of character scores by the number of characters. These calculations were performed using the *tidyverse* ‘rowSums’ function in R Statistical Software (v. 4.3.2) (R Core Team, 2023).

### Assessing correlation with prey capture method

We performed preliminary statistical tests to evaluate whether the suction-related character scores correlate with suction feeding. All analyses were performed in R Statistical Software (v. 4. 3. 2) (R Core Team, 2023), and the associated R scripts are provided in the Supplemental Information. We treated prey capture method as a categorical outcome with two levels (Suction *vs.* Other), and analyses were performed on all 68 taxa. Beaked whales, sperm whales, monodontids, *Globicephala*, and *Grampus griseus* were assigned “Suction”. This assignment was based on evidence independent of morphology (*e.g.*, experimental data, behavioral observations, stomach contents), as well as the presence of a significantly reduced dentition that necessitates capture suction feeding. Taxa assigned to “Other” include raptorial specialists and multimodal feeders, as well as species for which the distinction between raptorial and multimodal feeding suffers from a lack of observational data. For the analyses performed here, the crucial comparison is suction specialists *versus* species for which suction is not the primary method of prey capture (*i.e.,* specialized raptorial and multimodal feeding).

We performed Kruskal–Wallis rank sum tests to assess whether capture suction feeders (“Suction”) and raptorial or multimodal feeders (“Other”) significantly differ in character scores. Tests were conducted using the ‘kruskal.test’ function in the *stats* R package for each of the 10 suction-related characters, as well as both SSI values. However, species are non-independent data points, and the distribution of specialization scores might be explained by morphological similarity between closely related species. To measure phylogenetic signal in the specialization scores, we used a recent tree by Lloyd & Slater (2021) and the ‘phylosig’ function in the *phytools* R package to compute Blomberg’s *K* for each of the 10 morphological characters (Revell, 2024; Blomberg, Garland Jr & Ives, 2003). Because we found strong phylogenetic signal in the scores (Table S2), we used phylogenetic generalized least squares regression as an additional analysis that tests the correlation between prey capture method and specialization scores while accounting for phylogeny. For each of the 10 suction-related characters, we used the *nlme* function ‘gls’ (Pinheiro et al., 2025) to fit a linear model with expected covariance under Brownian motion (correlation = corBrownian), as well as a model with uncorrelated variance (Ordinary Least Squares, correlation = NULL) for comparison. We repeated the same analysis for SSI, as well as the adjusted SSI only accounting for osteological and dentition characters.

### Quantifying predictive power

We assessed the ability of individual and combined morphological traits to predict suction feeding in odontocetes using multinomial logistic regression. For each of the 10 suction-related characters, we trained separate single-variable models to evaluate their individual predictive value. We also tested the two composite measures, CharSum and CharMean, derived from the same ten traits. In all cases, the dataset was partitioned into training (75%) and testing (25%) subsets using stratified sampling to preserve class proportions. We evaluated the model performance on the test subset (25%) using standard evaluation metrics including Accuracy, Precision, F1-Score, and confusion matrices. Finally, we constructed a multivariate model incorporating all ten traits simultaneously. We performed multivariate analyses using the ‘multinom’ function in the R package *nnet*, with data partitioning and performance metrics provided by *caret* (R v. 4.3.2).

### Skull shape *vs.* suction specialization

To test whether suction specialization is associated with convergence in skull morphology, we conducted a principal component analysis (PCA) of 25 cranial and mandibular characters that capture skull shape. These characters and scorings follow recent phylogenetic matrices by Viglino et al. (2021), Meekin, Fordyce & Coste (2024) and Paolucci, Buono & Fernández (2025). These characters were specifically selected to be independent of our suction-related trait set. Scores for 30 of the 68 taxa were taken directly from the published matrices, and the remaining 38 taxa were manually coded from the literature. The PCA was performed on standardized character values using the ‘prcomp’ function in base R v. 4.3.2. Prey capture method (Suction and Other) and SSI values (CharSum, CharMean) were subsequently mapped onto the resulting morphospace to visualize their distribution. We projected phylogeny using the ‘phylomorphospace’ function in *phytools* v. 2.3-0 (Revell, 2024), with a recent tree by Lloyd & Slater (2021) as the phylogenetic framework.

To quantify the amount of convergence in skull shape among specialized suction feeders, we used C1 metric developed by Stayton (2015). C1 is calculated by dividing the distance between taxa in the morphospace by the maximum distance between ancestral nodes and subtracting the resulting quotient from 1 (Stayton, 2015). A C1 value that is close to 1 would indicate convergence on skull shape. We calculated C1 using all PCA axes (100% of the variation), and we assessed the value’s significance by running 1,000 Brownian motion simulations of character evolution using the ‘convSig’ function in *convevol* v. 2.2.1 (Brightly & Stayton, 2024; Zelditch et al., 2017).

## Results

**Figure 2:**
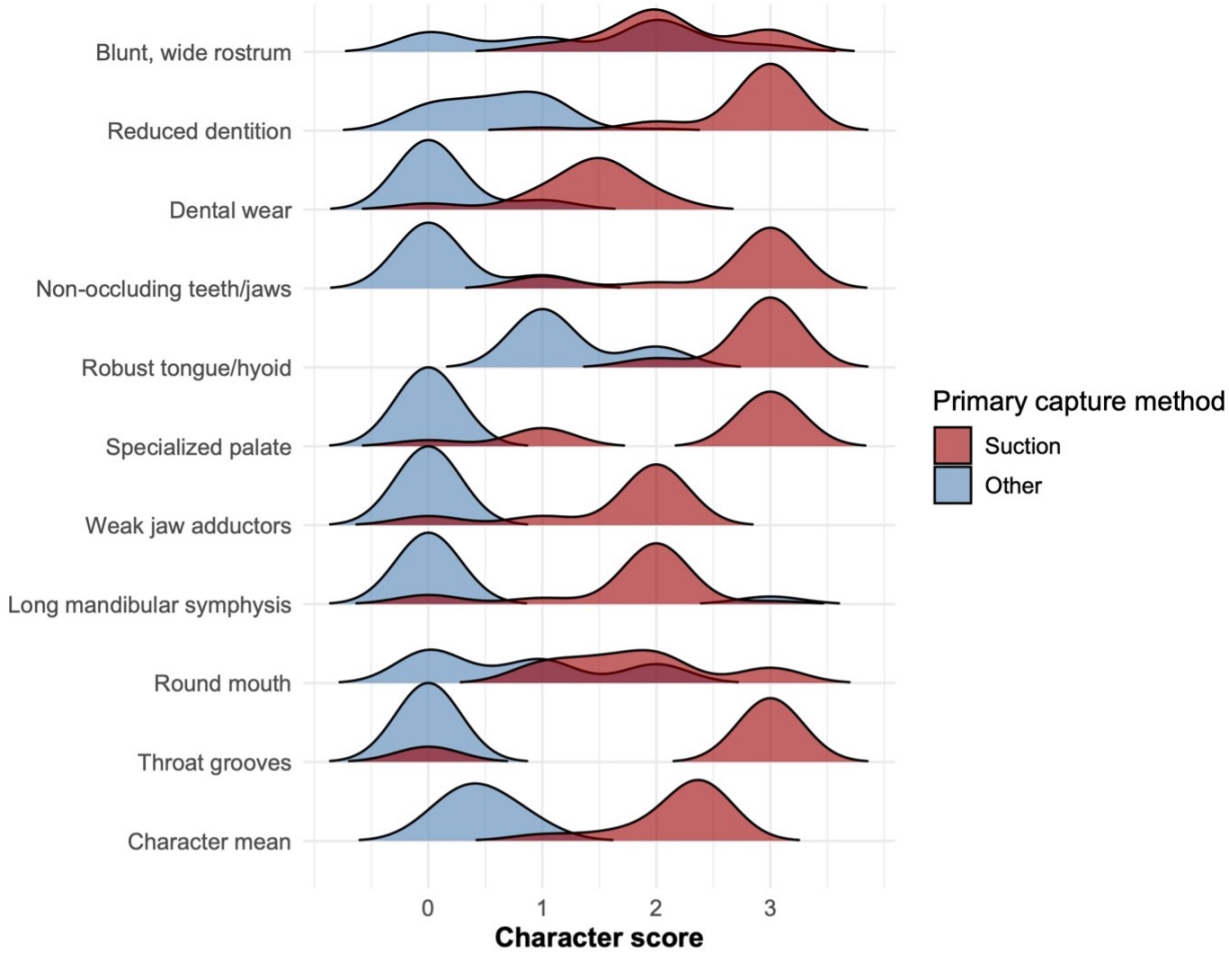
Distribution of suction-related character scores. Ridgeline plots showing the distribution of prey capture method (“primarily suction” *vs.* “primarily other”) across 10 suction-related characters, as well as mean character score. For each plot, the *x*-axis represents character score (0–3), and the *y*-axis represents frequency. Color indicates prey capture method.

We scored 68 extant cetacean taxa for 10 morphological characters related to suction feeding: blunt, wide rostrum, reduced dentition, dental wear, non-occluding teeth/jaws, robust tongue/hyoid, specialized palate, weak jaw adductors, long mandibular symphysis, round mouth, and throat grooves. Kruskal–Wallis tests indicate that capture suction feeders significantly differ from the rest of taxa with respect to each character (Fig. 2, Table S1). The phylogenetic least squares regression results also support strong correlations between capture suction feeding and the morphological characters, with exceptions in dental wear (*p* = 0.0918) and long mandibular symphysis (*p* = 0.6825) (Table S4). These results indicate that the scores of these two characters are not significantly correlated with suction feeding when phylogeny is taken into account.

Reduced dentition, non-occluding teeth/jaws, specialized palate, and weak jaw adductors were the strongest predictors (1.0 accuracy) (Table 1). Dental wear, robust tongue/hyoid, and throat grooves were also strong predictors (0.9375 accuracy), as well as long mandibular symphysis (0.8125 accuracy). Round mouth and blunt, wide rostrum were the weakest predictors (0.6875 and 0.50 accuracies, respectively). A multinomial regression model incorporating all 10 characters predicted suction with 0.94 accuracy. The model predicted suction with 1.0 accuracy when soft tissue characters (round mouth and throat grooves) were excluded.

**Table 1:** Single-variable and multinomial logistic regression results. The table lists accuracy values, ninety-five percent confidence intervals (95% CI), and *p*-values, where accuracy is greater than the no information rate (NIR = 0.625). Single-variable model results are reported for the 10 morphological predictors, as well as both versions of the suction specialization index (SSI). The table also reports the results of a multivariate model that incorporates all 10 morphological predictors simultaneously, as well as a multivariate model that only takes the eight osteological and dentition characters into account. Significant *p*-values (*p* < 0.05) are shown in bold.

**Predictor(s)**	**Accuracy**	**95% CI**	***p*** **-value**
Blunt, wide rostrum	0.5	(0.2465, 0.7535)	0.9001
Reduced dentition	1	**(**0.7941, 1)	**0.0005421**
Dental wear	0.9375	(0.6977, 0.9984)	**0.005746**
Non-occluding teeth/jaws	1	(0.7941, 1)	**0.0005421**
Robust tongue/hyoid	0.9375	(0.6977, 0.9984)	**0.005746**
Specialized palate	1	(0.7941, 1)	**0.0005421**
Weak jaw adductors	1	(0.7941, 1)	**0.0005421**
Long mandibular symphysis	0.8125	(0.5435, 0.9595)	0.09474
Round mouth	0.6875	(0.4134, 0.8898)	0.4067
Throat grooves	0.9375	(0.6977, 0.9984)	**0.005746**
SSI (all 10 morphological characters)	0.9375	(0.6977, 0.9984)	**0.005746**
Adjusted SSI (osteology and dentition characters only)	1	(0.7941, 1)	**0.0005421**
All 10 morphological characters (multivariate model)	0.9375	(0.6977, 0.9984)	**0.005746**
Eight osteological and dentition characters only (multivariate model)	1	(0.7941, 1)	**0.0005421**

We quantified overall suction specialization by calculating the mean character score for each taxon. Here, we refer to this value as the SSI. The SSI was significantly different for capture suction feeders (*p* < 0.05) and had high predictive power (0.9375 accuracy). The SSI only accounting for osteological and dentition characters also had high predictive power (1.0 accuracy).

*Ziphius cavirostris* scored the highest in overall suction specialization (SSI= 2.55, SD = 0.497). *Delphinus* and *Stenella* were the least specialized for suction (SSI = 0.1, SD = 0.316). Sperm whales, most beaked whales, and *Monodon monoceros* scored at or above an SSI of 2.00. *Delphinapterus leucas, Grampus griseus,* and *Tasmacetus shepherdi* scored at or above an SSI of 1.50. *Globicephala* and *Orcaella brevirostris* scored at or above an SSI of 1.00.

**Figure 3:**
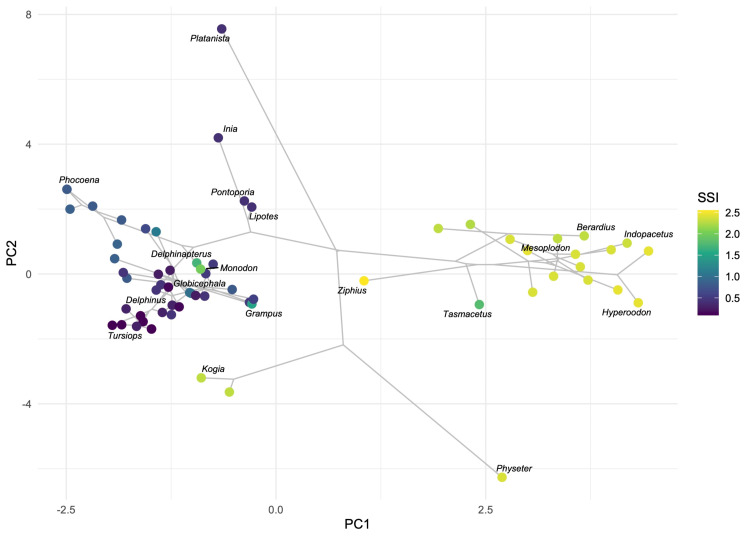
Specialized suction feeders do not converge on skull shape. The resulting morphospace of the PCA on skull characters, with the suction specialization index (SSI) superimposed. PC1 (18.4% of variation) and PC2 (13.1% of variation) are plotted on the *x*- and *y*-axes, representing a total of 31.5% of the variation in the data. Color indicates SSI. Phylogeny was projected using a recent tree by Lloyd & Slater (2021).

We performed a principal component analysis on 25 cranial and mandibular characters that capture skull shape. The first two PC’s accounted for 31.5% of the total variation in the data (PC1 = 18.4% and PC2 = 13.1%). The third and fourth PC’s accounted for 20.7% of the total variation (PC3 = 12.1% and PC4 = 8.6%) (Fig. S1). The rest of the PCA results are listed in Table S4 in the Supplemental Information. When PC1 and PC2 are plotted on the *x*- and *y*-axes, suction specialists (species with the highest SSI) clearly occupy different areas of the morphospace (Fig. 3). The C1 metric (C1 = 0.0110, *p* = 0.862) also suggests no significant convergence in skull shape.

## Discussion

Here, we tested the inference of suction feeding from a suite of morphological predictors. Model accuracy, as well as the strength of individual predictors, demonstrate that morphological evidence can provide a robust foundation for identifying suction feeders among odontocetes. Perfect accuracy of the model accounting only for osteological correlates and dentition indicates that suction can be reasonably inferred without direct observation of soft tissue structures (as in fossils).

Of the 10 morphological characters examined in this study, blunt, wide rostrum was the weakest predictor. This result is surprising, given the attention short, blunt rostra (and wide mandibles) have received as adaptations for suction feeding (Werth, 2006b; Werth, 2006a; Boessenecker et al., 2017). Short, wide rostra are common among kogiids and suction-feeding delphinoids (Werth, 2006a; Galatius et al., 2020). A blunt rostrum may enhance suction performance by improving water flow towards the mouth and contributing to a rounder oral opening (Werth, 2006b). However, other structures may enhance suction performance in taxa with less blunt skull profiles. In *Physeter*, a round opening may be accomplished by the oropharyngeal isthmus located at the entrance of the pharynx (Werth, 2004). Larger body sizes in *Physeter* and beaked whales may also minimize the importance of a blunt rostrum for drawing sufficient amounts of water into the oral cavity (McCurry et al., 2017a).

“Obligate” suction feeders, such as beaked whales and sperm whales, had the highest overall specialization scores. These taxa strongly exhibit traits that facilitate suction generation and performance (*e.g.*, robust tongue/hyoid), as well as those that impose severe constraints on feeding mode (*e.g.*, reduced dentition). Less specialized suction feeders, such as *Globicephala,* may still be capable in engaging in raptorial behavior (Werth, 2000b; Kane & Marshall, 2009). Lower specialization scores in *Globicephala* and *Grampus* may also suggest that capture suction feeding evolved recently in delphinids. The assumption of suction feeding in *Orcaella brevirostris* is supported by similar scores, as well as observations of water “spitting” behavior (Marsh et al., 1989).

Although low specialization scores reflect less confidence in asserting capture suction feeding, they do not rule out the possibility of suction-feeding behavior. We examined only 10 suction-related characters, but there may be other useful morphological predictors for suction feeding (*e.g.*, muscle attachment scars on the mandible (Strauch, Pyenson & Peredo, 2025). Porpoises exhibit few of the morphological specializations examined in this study, yet some experimental data suggests they utilize suction for prey capture (Kastelein et al., 1997; Werth, 2006b). In Dall’s porpoise (*Phocoenoides dalli*), the tooth row is buried by gingiva (Cowan, 1944). Suction may be more likely in this species than our scores suggest. For killer whales, robust hyoids and blunt, wide jaws—as well as behavioral observations—indicate that these grip-and-tear feeders are capable of generating and employing suction (Werth, 2006a; Donaldson, 1977). Species that opportunistically use both raptorial and suction prey-capture techniques might be characterized as multimodal or generalist feeders. While we only focus on suction-related traits here, future work that evaluates morphological traits associated with other feeding modes might further delimit generalized and specialized morphologies.

Numerous studies have investigated the ecomorphology of the cetacean skull (McCurry et al., 2017b; Galatius et al., 2020; Coombs et al., 2024; McCurry et al., 2017a; Coombs et al., 2022; Vicari et al., 2023; Gol’din & Vishnyakova, 2016; McCurry et al., 2023). However, these analyses are limited by a lack of observational data on feeding behavior (Coombs et al., 2022). Feeding strategy (*e.g.*, suction feeding) is typically treated as a categorial variable, with each taxon assigned to a single category based on the assumed predominant feeding mode. These categories are often challenging to define and do not reflect the fact that behavior may fall along a continuum (Hocking et al., 2017; Carrano, 1999). Our solution to this problem was to use the extent of morphological specialization (SSI) as a proxy for a particular mode of feeding (*i.e.,* capture suction feeding).

The PCA results demonstrate that suction specialization does not coincide with a specific skull shape. Suction-feeding lineages (*i.e.,* beaked whales, sperm whales, monodontids, and some delphinids) do not specialize towards the same area of the morphospace (Fig. 3). While suction-feeding taxa converge on several morphological traits, skull shape is likely constrained by phylogeny. Skull shape may also be influenced by prey size, habitat, and latitude (McCurry et al., 2017b; Gol’din & Vishnyakova, 2016; McCurry et al., 2023). Although skull shape strongly correlates with other modes of feeding (McCurry et al., 2017b; McCurry et al., 2017a), diverse skull shapes across suction feeders might reflect relaxed and/or different selective pressures.

## Conclusions

The parallel evolution of specialized suction feeding in odontocetes exemplifies specialization without wholesale convergence on form. So-called “incomplete convergence” on morphology is likely a common phenomenon in nature (Grossnickle et al., 2020), with other examples of limited morphological convergence in the evolution of cursoriality in tetrapods, myrmecophagy in mammals, and endothermy in fish (Carrano, 1999; Vida, Calamari & Barden, 2025; Melendez-Vazquez et al., 2025). It is possible that suction feeding in cetaceans is a true case of many-to-one mapping, in which different morphologies produce the same functional outcome (Wainwright et al., 2005). Future work should aim to measure and compare performance across different suction-feeding morphologies. The application of SSI to fossil taxa might further illuminate the evolution of suction feeding in toothed whales by quantifying change in the degree of specialization within a lineage through geologic time. For ecological reconstructions, SSI values greater than 1.00 might be considered strong evidence for specialized suction feeding in fossil odontocetes for which all eight of the osteological and dentition characters are preserved. Our approach—testing the strength of morphological predictors and quantifying the degree of specialization—also provides a framework that might be applied to other taxa, especially extinct species for which behavior cannot be directly observed.

## Supplemental Information

10.7717/peerj.21119/supp-1 Supplemental Information 1 Data and R scripts used to perform logistic regression and principal component analyses

10.7717/peerj.21119/supp-2 Supplemental Information 2 List of taxa and suction scores, PCA results, and list of cranial and mandibular characters

## Additional Information and Declarations

### Data Availability

The following information was supplied regarding data availability: The data and code used to perform phylogenetic least squares regression, logistic regression, and principal component analyses are available in the Supplemental Files.
