# Supplementary material for: Toothed whales independently evolve specialized suction feeding without converging on skull shape
Source: PeerJ. 2026 Apr 17;14:e21119. doi: 10.7717/peerj.21119 (PMC13094552; doi:10.7717/peerj.21119)
Supplement: Supplemental Information 2 [file peerj-14-21119-s002.pdf]

Supplemental Information

List of suction-related morphological characters

Table S1: List of taxa and suction scores

Table S2: Phylogenetic signal in suction scores

Table S3: Phylogenetic generalized least squares results

Table S4: PCA results

Figure S1: Morphospace of PC3 and PC4

List of cranial and mandibular characters

List of suction-related morphological characters

1. **Blunt, wide rostrum**

In odontocetes, blunt head shape contributes to creating a round oral aperture and limiting lateral ("notched") gape [1–2]. Experimental data suggests that species with blunt head shapes generate greater negative pressures [1]. Blunt head shape is also related to "amblygnathy" (shortened, rounded jaws and rostra) [2]. In odontocetes, "amblygnathy" can be quantified by the mandibular bluntness index (MBI)—a dimensionless ratio of mandibular width:length. MBI strongly correlates with dental reduction in toothed whales [2].

Scoring reflects the degree to which the trait is manifested (3=strongly present/highly apparent, 2=somewhat present, 1=weakly present, 0=never present).

Examples:
Globicephala macrorhynchus (short-finned pilot whale): 3
Ziphius cavirostris (Cuvier's beaked whale): 2
Tursiops truncatus (common bottlenose dolphin): 1
Stenella longirostris (long snouted spinner dolphin): 0

2. **Reduced dentition**

Suction-feeding taxa may use teeth to secure prey, but not to ingest prey via biting or seizing [2]. Reduced dentition may be interpreted in several ways, including reduction in number of teeth, size of erupted crown, complexity of crown, and "functionality" of the teeth for seizing prey (i.e., protruding above the mouth in beaked whales) [2–4].

Scoring reflects the degree to which the trait is manifested (3=strongly present/highly apparent, 2=somewhat present, 1=weakly present, 0=never present).

Examples:
Monodon monoceros (narwhal): 3
Delphinapterus leucas (beluga): 2
Phocoena phocoena (harbor porpoise): 1
Platanista gangetica (Ganges river dolphin): 0

3. **Dental wear**

During suction feeding, teeth can incur distinct patterns of wear via water flow, abrasion, and movements of the tongue [5]. The severity of tooth wear may also reflect the softness and/or thinness of the enamel, which in turn relates to the unimportance of the dentition in capturing prey [6].

Scoring reflects the degree to which the trait is manifested (2=strongly present/highly apparent, 1=somewhat present, 0=weakly or never present).

Examples:
Delphinapterus leucas (beluga): 2
Kogia breviceps (pygmy sperm whale): 1
Stenella longirostris (long snouted spinner dolphin): 0

4. **Non-occluding teeth/jaws**

Typical raptorial feeding may be inefficient if the teeth and jaws do not occlude. Teeth cannot occlude in extreme cases of dental reduction, such as the lack of erupted maxillary teeth (as in sperm whales and *Grampus*) [2]. Adult specimens with jaws that are congenitally curved—or broken and re-healed at angles precluding occlusion—suggest the unimportance of functional teeth and jaws for seizing prey [7].

Scoring reflects the degree to which the trait is manifested (3=strongly present/highly apparent, 2=somewhat present, 1=weakly present, 0=never present).

Examples:
Mesoplodon layardii (strap-toothed whale): 3
Kogia breviceps (pygmy sperm whale): 2
Delphinapterus leucas (beluga): 1
Stenella longirostris (long snouted spinner dolphin): 0

5. **Robust tongue/hyoid**

In odontocetes, intraoral suction is generated by rapid, piston-like depression and retraction of the tongue [8]. Extrinsic muscles involved in depressing and retracting the tongue originate on the hyoid skeleton. An enlarged hyoid skeleton, with robust thyrohyal and stylohyal elements, indicate ample attachment sites for prominent tongue muscles [8–10].

Scoring reflects the degree to which the trait is manifested (3=strongly present/highly apparent, 2=somewhat present, 1=weakly present).

Examples:
Physeter macrocephalus (giant sperm whale): 3
Globicephala macrorhynchus (short-finned pilot whale): 2
Tursiops truncatus (common bottlenose dolphin): 1

6. **Specialized palate**

Palates can be specialized in at least two ways for suction feeding. First, vaulted palates can hold a large tongue and ingested water (as well as prey) [3, 11–12]. Second, rough palatal surfaces—

such as cornified keratinous ridges in beaked whales—can hold slippery prey (e.g., cephalopods) in the absence of functional dentition for grasping [3].

Scoring reflects the degree to which the trait is manifested (3=strongly present/highly apparent, 1=somewhat present, 0=never present).

Examples:
Ziphius cavirostris (Cuvier's beaked whale): 3
Delphinapterus leucas (beluga): 1
Tursiops truncatus (common bottlenose dolphin): 0

7. **Weak jaw adductors**

Strong jaw-closing muscles (such as the temporalis muscle) are important for capturing prey via biting or seizing [13–14], but less so for suction ingestion. In fossils, muscle attachment sites (such as the temporal fossa) are useful osteological correlates for the size and importance of jaw adductors [14–15].

Scoring reflects the degree to which the trait is manifested (2=strongly present/highly apparent, 1=somewhat present, 0=never present).

Example:
Ziphius cavirostris (Cuvier's beaked whale): 2
Delphinapterus leucas (beluga): 1
Orcinus orca (killer whale): 0

8. **Long mandibular symphysis**

An elongated mandibular symphysis indicates an atypical oral cavity in which the tongue lies partially or entirely behind (rather than between) the tooth rows [7–8]. The extreme caudal position of the tongue in some suction feeders—such as *Physeter macrocephalus* (giant sperm whales)—suggests that prey are likely sucked directly into the oropharynx [7]. This also suggest that teeth (if present) are likely used for retaining prey, rather than prey prehension or transport [16].

Scoring reflects the degree to which the trait is manifested (3=strongly present/highly apparent, 2=somewhat present, 1=weakly present, 0=never present).

Example:
Physeter macrocephalus (giant sperm whale): 3
Ziphius cavirostris (Cuvier's beaked whale): 2
Delphinapterus leucas (beluga): 1
Phocoena phocoena (harbor porpoise): 0

9. **Round mouth**

A rounder oral aperture improves incurrent flow for suction ingestion, as opposed to a notched "Pac-Man" gape with open sides [1, 10]. Some suction feeders, such as belugas (*Delphinapterus leucas*), are capable of pursing the oral aperture into a circular shape [17]. In giant sperm whales (*Physeter macrocephalus*), the round aperture may be accomplished by the oropharyngeal isthmus [7]. Because a round oral aperture is usually dictated by soft-tissue orofacial morphology, this trait is likely undetectable in fossils.

Scoring reflects the degree to which the trait is manifested (3=strongly present/highly apparent, 2=somewhat present, 1=weakly present, 0=never present).

Example:
Kogia breviceps (pygmy sperm whale): 3
Mesoplodon layardii (strap toothed beaked whale): 2
Lagenorhynchus obscurus (dusky dolphin): 1
Stenella longirostris (long snouted spinner dolphin): 0

10. **Throat grooves**

Throat grooves allow for gular expansion to accommodate the influx of water during suction ingestion of water and prey [3].

Scoring reflects presence or absence (3=present, 0=absent).

Examples:
Ziphius cavirostris (Cuvier's beaked whale): 3
Globicephala macrorhynchus (short-finned pilot whale): 0

References

1. Werth AJ. 2006 Odontocete suction feeding: experimental analysis of water flow and head shape. *J. Morph.* **267**, 1415–1428. (doi:10.1002/jmor.10486)
2. Werth AJ. 2006 Mandibular and dental variation and the evolution of suction feeding in Odontoceti. *J. Mamm.* **87**, 579–588. (doi:10.1644/05-MAMM-A-279R1.1)
3. Heyning JE, Mead JG. 1996 Suction feeding in beaked whales: morphological and observational evidence. *Contrib. Sci., Nat. Hist. Mus. Los Angeles County* **464**, 1–12.
4. Loch C, Fordyce RE, Werth AJ. 2023 Skulls, teeth, and sex. In *Sex in Cetaceans: Morphology, Behavior, and the Evolution of Sexual Strategies* (eds Würsig B, Orbach DN), pp. 51–64. Cham, Switzerland: Springer.
5. Marx FG, Hocking DP, Park T, Pollock TI, Parker WM, Rule JP, Fitzgerald EMG, Evans AR. 2023 Suction causes novel tooth wear in marine mammals, with implications for feeding evolution in baleen whales. *J. Mamm. Evol.* **30**, 493–505. (doi:https://doi.org/10.1007/s10914-022-09645-1)

6. Werth AJ, Loch C, Fordyce RE. 2020 Enamel microstructure in Cetacea: a case study in evolutionary loss of complexity. *Journal of Mammalian Evolution* **27**, 789–805. (doi:https://doi.org/10.1007/s10914-019-09484-7)
7. Werth AJ. 2004 Functional morphology of the sperm whale (*Physeter macrocephalus*) tongue, with reference to suction feeding. *Aquat. Mamm.* **30**, 405–418. (doi:10.1578/AM.30.3.2004.405)
8. Werth AJ. 2007 Adaptations of the cetacean hyolingual apparatus for aquatic feeding and thermoregulation. *Anat. Rec.* **290**, 546–568. (doi:10.1002/ar.20538)
9. Reidenberg JS, Laitman JT. 1994 Anatomy of the hyoid apparatus in Odontoceti (toothed whales): specializations of their skeleton and musculature compared with those of terrestrial mammals. *Anat. Rec.* **240**, 598–624. (doi:10.1002/ar.1092400417)
10. Bloodworth B, Marshall CD. 2007 A functional comparison of the hyolingual complex in pygmy and dwarf sperm whales (*Kogia breviceps* & *K. sima*), and bottlenose dolphins (*Tursiops truncatus*). *J. Anat*. **211**, 78–91. (doi:10.1111/j.1469-7580.2007.00755.x)
11. Kastelein RA, Gerrits NM. 1990 The anatomy of the walrus head (*Odobenus rosmarus*). Part 1: The skull. *Aquat. Mamm.* **16**, 101–119.
12. Marshall CD, Kovacs KM, Lydersen C. 2008 Feeding kinematics, suction and hydraulic jetting capabilities in bearded seals (*Erignathus barbatus*). *J. Exp. Biol.* **211**, 699–708. (doi:10.1242/jeb.009852)
13. Seagars DJ. 1982 Jaw structure and functional mechanics of six delphinids (Cetacea: Odontoceti). Master's thesis, San Diego State University.
14. Werth AJ, Beatty BL. 2023 Osteological correlates of evolutionary transitions in cetacean feeding and related oropharyngeal functions. *Front. Ecol. Evol.* **11**, 1179804. (doi:10.3389/fevo.2023.1179804)
15. Xiong DC, Beatty BL, Churchill M. 2024 Temporalis attachment area as a proxy for feeding ecology in toothed whales (Artiodactyla: Odontoceti). *PaleoBios* **41**, 1–29. (doi:https://doi.org/10.5070/P941361509)
16. Werth AJ, Crompton AW. 2023 Cetacean tongue mobility and function: A comparative review. *J. Anat.* **243**, 343–373. (doi:https://doi.org/10.1111/joa.13876)
17. Kane EA, Marshall CD. 2009 Comparative feeding kinematics and performance of odontocetes: belugas, Pacific white-sided dolphins and long-finned pilot whales. *J. Exp. Biol.* **212**, 3939–3950. (doi:10.1242/jeb.034686)

TABLE S1

Suction scores based on morphological characters A-J: A = Round mouth (H(1) = 20.1, p < 0.001), B = Throat grooves (H(1) = 48.4, p < 0.001), C = Blunt/wide rostrum (H(1) = 9.10, p = 0.003), D = Reduced dentition (H(1) = 48.7, p < 0.001), E = Dental wear (H(1) = 47.3, p < 0.001), F = Non-occluding teeth/jaws (H = 54.0, p < 0.001), G = Robust tongue/hyoid (H(1) = 52.0, p < 0.001), H = Specialized palate (H(1) = 57.0, p < 0.001), I = Weak jaw adductors (H(1) = 54.3, p < 0.001), J = Long mandibular symphysis (H(1) = 31.6, p < 0.001). Character sum, average, and standard deviation are reported for the entire suite of characters (soft tissue + osteology), as well as the osteological and dentition characters only.

Species	Morphological characters										Characters A-J			Characters C-J		
	Soft Tissue		Osteology and Dentition													
	A	B	C	D	E	F	G	H	I	J	Sum	Avg.	SD	Sum	Avg.	SD
Aethalodelphis obliquidens	1	0	2	0.5	0	0	1	0	0	0	4.5	0.45	0.685	3.5	0.438	0.729
Aethalodelphis obscurus	1	0	2	0.5	0	0	1	0	0	0	4.5	0.45	0.685	3.5	0.438	0.729
Berardius arnuxii	1	3	1.5	3	1.5	3	3	3	2	2	23	2.3	0.789	19	2.375	0.694
Berardius bairdii	1	3	1.5	3	1.5	3	3	3	2	2	23	2.3	0.789	19	2.375	0.694
Cephalorhynchus australis	1	0	2	0.5	0	0	1	0	0	0	4.5	0.45	0.685	3.5	0.438	0.729
Cephalorhynchus commersonii	1	0	2	1	0	0	1	0	0	0	5	0.5	0.707	4	0.500	0.756
Cephalorhynchus cruciger	1	0	2	0.5	0	0	1	0	0	0	4.5	0.45	0.685	3.5	0.438	0.729
Cephalorhynchus eutropia	1	0	2	1	0	0	1	0	0	0	5	0.5	0.707	4	0.500	0.756
Cephalorhynchus heavisidii	1	0	2	1	0	0	1	0	0	0	5	0.5	0.707	4	0.500	0.756
Cephalorhynchus hectori	1	0	2	1	0	0	1	0	0	0	5	0.5	0.707	4	0.500	0.756
Delphinapterus leucas	3	0	3	2	2	1	3	1	1	1	17	1.7	1.059	14	1.750	0.886
Delphinus delphis	0	0	0	0	0	0	1	0	0	0	1	0.1	0.316	1	0.125	0.354
Feresa attenuata	2	0	3	1	0	0	2	0	0	0	8	0.8	1.135	6	0.750	1.165
Globicephala macrorhynchus	2	0	3	2	0	1	2	0	0	0	10	1	1.155	8	1.000	1.195
Globicephala melas	2	0	3	2	0	1	2	0	0	0	10	1	1.155	8	1.000	1.195
Grampus griseus	2	0	3	3	1	3	2	1	0	0	15	1.5	1.269	13	1.625	1.302
Hyperoodon ampullatus	2	3	2	3	1.5	3	3	3	2	2	24.5	2.45	0.599	19.5	2.438	0.623
Hyperoodon planifrons	2	3	2	3	1.5	3	3	3	2	2	24.5	2.45	0.599	19.5	2.438	0.623
Indopacetus pacificus	1	3	2	3	1.5	3	3	3	2	2	23.5	2.35	0.747	19.5	2.438	0.623
Inia geoffrensis	0	0	0	0	0	0	1	0	0	3	4	0.4	0.966	4	0.500	1.069
Kogia breviceps	3	3	3	3	1	2	3	1	2	2	23	2.3	0.823	17	2.125	0.835
Kogia sima	3	3	3	3	1	2	3	1	2	2	23	2.3	0.823	17	2.125	0.835
Lagenodelphis hosei	1	0	2	0.5	0	0	1	0	0	0	4.5	0.45	0.685	3.5	0.438	0.729

Lagenorhynchus albirostris	1	0	2	0.5	0	0	1	0	0	0	4.5	0.45	0.685	3.5	0.438	0.729
Leucopleurus acutus	1	0	2	0.5	0	0	1	0	0	0	4.5	0.45	0.685	3.5	0.438	0.729
Lipotes vexillifer	0	0	0	0	0	0	1	0	0	3	4	0.4	0.966	4	0.500	1.069
Lissodelphis borealis	0.5	0	1	0.5	0	0	1	0	0	0	3	0.3	0.422	2.5	0.313	0.458
Lissodelphis peronii	0.5	0	1	0.5	0	0	1	0	0	0	3	0.3	0.422	2.5	0.313	0.458
Mesoplodon bidens	1	3	1.5	3	1.5	3	3	3	2	2	23	2.3	0.789	19	2.375	0.694
Mesoplodon bowdoini	1.5	3	2	3	1.5	3	3	3	2	2	24	2.4	0.658	19.5	2.438	0.623
Mesoplodon carlhubbsi	1.5	3	2	3	1.5	3	3	3	2	2	24	2.4	0.658	19.5	2.438	0.623
Mesoplodon densirostris	2	3	2	3	1.5	3	3	3	2	2	24.5	2.45	0.599	19.5	2.438	0.623
Mesoplodon europaeus	1.5	3	2	3	1.5	3	3	3	2	2	24	2.4	0.658	19.5	2.438	0.623
Mesoplodon grayi	1	3	1	3	1.5	3	3	3	2	2	22.5	2.25	0.858	18.5	2.313	0.799
Mesoplodon hectori	1.5	3	2	3	1.5	3	3	3	2	2	24	2.4	0.658	19.5	2.438	0.623
Mesoplodon layardii	2	3	2	3	1	3	3	3	2	2	24	2.4	0.699	19	2.375	0.744
Mesoplodon mirus	1.5	3	2	3	1.5	3	3	3	2	2	24	2.4	0.658	19.5	2.438	0.623
Mesoplodon peruvianus	1.5	3	2	3	1.5	3	3	3	2	2	24	2.4	0.658	19.5	2.438	0.623
Mesoplodon stejnegeri	2	3	2	3	1.5	3	3	3	2	2	24.5	2.45	0.599	19.5	2.438	0.623
Monodon monoceros	3	0	3	3	2	3	3	1	1	1	20	2	1.155	17	2.125	0.991
Neophocaena phocaenoides	2	0	3	1	0	1	2	0	0	0	9	0.9	1.101	7	0.875	1.126
Orcaella brevirostris	2	0	3	2	1	1	2	0	0	0	11	1.1	1.101	9	1.125	1.126
Orcinus orca	0.5	0	2	1	1	0	2	0	0	0	6.5	0.65	0.818	6	0.750	0.886
Peponocephala electra	2	0	2	1	1	0	2	0	0	0	8	0.8	0.919	6	0.750	0.886
Phocoena dioptrica	2	0	2.5	1	0	1	2	0	0	0	8.5	0.85	1.001	6.5	0.813	0.998
Phocoena phocoena	2	0	2	1	0	1	2	0	0	0	8	0.8	0.919	6	0.750	0.886
Phocoena sinus	2	0	2	1	0	1	2	0	0	0	8	0.8	0.919	6	0.750	0.886
Phocoena spinnipinnis	2	0	2	1	0	1	2	0	0	0	8	0.8	0.919	6	0.750	0.886
Phocoenoides dalli	2	0	2.5	1	0	1	2	0	0	0	8.5	0.85	1.001	6.5	0.813	0.998
Physeter macrocephalus	3	3	2	3	2	3	3	1	1	3	24	2.4	0.843	18	2.250	0.886
Platanista gangetica	0	0	0	0	0	0	1	0	0	3	4	0.4	0.966	4	0.500	1.069
Pontoporia blainvillei	0	0	0	0	0	0	1	0	0	3	4	0.4	0.966	4	0.500	1.069
Pseudorca crassidens	2	0	2.5	1	1	0	2	0	0	0	8.5	0.85	1.001	6.5	0.813	0.998
Sotalia fluviatilis	0	0	0.5	0	0	0	1	0	0	0	1.5	0.15	0.337	1.5	0.188	0.372
Sotalia guianensis	0	0	0.5	0	0	0	1	0	0	0	1.5	0.15	0.337	1.5	0.188	0.372
Sousa chinensis	0	0	1	0.5	0	0	1	0	0	0	2.5	0.25	0.425	2.5	0.313	0.458

Sousa plumbea	0	0	1	0.5	0	0	1	0	0	0	2.5	0.25	0.425	2.5	0.313	0.458
Sousa teuszii	0	0	1	0.5	0	0	1	0	0	0	2.5	0.25	0.425	2.5	0.313	0.458
Stenella attentuata	0	0	0	0	0	0	1	0	0	0	1	0.1	0.316	1	0.125	0.354
Stenella clymene	0	0	0	0	0	0	1	0	0	0	1	0.1	0.316	1	0.125	0.354
Stenella coeruleoalba	0	0	0	0	0	0	1	0	0	0	1	0.1	0.316	1	0.125	0.354
Stenella frontalis	0	0	0	0	0	0	1	0	0	0	1	0.1	0.316	1	0.125	0.354
Stenella longirostris	0	0	0	0	0	0	1	0	0	0	1	0.1	0.316	1	0.125	0.354
Steno bredanensis	1	0	1.5	1	1	0	1	0	0	0	5.5	0.55	0.599	4.5	0.563	0.623
Tasmacetus shepherdi	1	3	1	1	1	1	3	3	2	2	18	1.8	0.919	14	1.750	0.886
Tursiops aduncus	0	0	1	1	0	0	1	0	0	0	3	0.3	0.483	3	0.375	0.518
Tursiops truncatus	0	0	1	1	0	0	1	0	0	0	3	0.3	0.483	3	0.375	0.518
Ziphius cavirostris	2	3	2.5	3	2	3	3	3	2	2	25.5	2.55	0.497	20.5	2.563	0.496

TABLE S2

Phylogenetic signal in the suction-related character scores. The table lists Bloomberg's *K* and *p*-value for each morphological character, as well as the suction specialization index (SSI). Results for SSI are reported for both the index calculated from all 10 characters (soft tissue +osteology) and the adjusted index (osteology only). Larger *K* values indicate stronger phylogenetic signal; *p*-values indicate that the *K* values significantly differ from zero.

**Morphological character**	**Bloomberg's *K***	***p*-value**
**Blunt, wide rostrum**	0.65403	**<0.001**
**Reduced dentition**	1.37426	**<0.001**
**Dental wear**	1.69405	**<0.001**
**Non-occluding teeth/jaws**	1.88985	**<0.001**
**Robust tongue/hyoid**	3.66575	**<0.001**
**Specialized palate**	7.16255	**<0.001**
**Weak jaw adductors**	8.88597	**<0.001**
**Long mandibular symphysis**	13.3135	**<0.001**
**Round mouth**	1.35863	**<0.001**
**Throat grooves**	11.5185	**<0.001**
**SSI (soft tissue + osteology)**	4.47371	**<0.001**
**SSI (osteology only)**	**4.47371**	**<0.001**

TABLE S3

Results of phylogenetic generalized least squares regression testing the correlation between morphology and prey capture method. The table reports the results of two linear models (Brownian motion, BM, Ordinary Least Squares, OLS) for each morphological character, as well as the suction specialization index (SSI). Results for SSI are reported for both the index calculated from all 10 characters (soft tissue +osteology) and the adjusted index (osteology only). Akaike criterion values (AIC), coefficients, standard errors of coefficient (SE), *t*-values, and *p*-values are listed.

**Morphological character**	**Model**	**AIC**	**Coefficient**	**SE**	***t*-value**	***p*-value**
**Blunt, wide rostrum**	BM	125.1027	0.8977	0.3233	2.7767	**0.0071**
	OLS	176.4000	0.7729	0.2146	3.6024	**<0.001**
**Reduced dentition**	BM	79.5226	1.6168	0.2312	6.9922	**<0.001**
	OLS	96.9069	2.2125	0.1196	18.5007	**<0.001**
**Dental wear**	BM	42.1662	0.3006	0.1757	1.7110	0.0918
	OLS	73.1214	1.2463	0.1004	12.4138	**<0.001**
**Non-occluding teeth/jaws**	BM	56.9968	1.8022	0.1959	9.1981	**<0.001**
	OLS	115.7153	2.4487	0.1373	17.8317	**<0.001**
**Robust tongue/hyoid**	BM	0.8052	0.5692	0.1296	4.3912	**<0.001**
	OLS	73.6874	1.6227	0.1008	16.0955	**<0.001**
**Specialized palate**	BM	-11.8839	0.7159	0.1181	6.0630	**<0.001**
	OLS	142.2763	2.3077	0.1669	13.8233	**<0.001**
**Weak jaw adductors**	BM	-53.042	0.3596	0.0872	4.1221	**<0.001**
	OLS	80.3658	1.6538	0.1059	15.6182	**<0.001**
**Long mandibular symphysis**	BM	7.5833	0.0560	0.1362	0.4109	0.6825
	OLS	171.9322	1.4451	0.2076	6.9601	**<0.001**
**Round mouth**	BM	74.4161	0.6736	0.2227	3.0245	**0.0035**
	OLS	159.2516	1.0485	0.1891	5.5438	**<0.001**
**Throat grooves**	BM	-0.2485	0.3699	0.1286	2.8759	**0.0054**
	OLS	156.3791	2.4231	0.1852	13.0848	**<0.001**
**SSI (soft tissue + osteology)**	BM	-27.5962	0.7361	0.1052	6.9983	**<0.001**
	OLS	48.9132	1.7181	0.0840	20.4471	**<0.001**
**SSI (osteology only)**	BM	2.7513	0.9202	0.1315	6.9983	**<0.001**
	OLS	79.2607	2.1477	0.1050	20.4471	**<0.001**

TABLE S4

Results of a principal component analysis on 25 cetacean skull characters. Characters and scorings are based on recent phylogenetic matrices by Viglino et al. (2021), Meekin et al. (2024), and Paolucci et al (2025). Principal components and their proportion of variance, standard deviation, and cumulative proportion are listed.

Principal Component	Proportion of Variance	Standard Deviation	Cumulative Proportion
PC1	18.39%	2.14	18.39%
PC2	13.11%	1.81	31.50%
PC3	12.08%	1.74	43.58%
PC4	8.63%	1.47	52.21%
PC5	7.34%	1.35	59.55%
PC6	5.75%	1.20	65.30%
PC7	4.98%	1.12	70.28%
PC8	4.25%	1.03	74.53%
PC9	4.21%	1.03	78.75%
PC10	3.33%	0.913	82.08%
PC11	2.78%	0.834	84.86%
PC12	2.23%	0.747	87.09%
PC13	2.21%	0.743	89.30%
PC14	1.94%	0.697	91.24%
PC15	1.77%	0.665	93.01%
PC16	1.52%	0.616	94.53%
PC17	1.36%	0.583	95.89%
PC18	1.03%	0.507	96.92%
PC19	0.89%	0.472	97.81%
PC20	0.72%	0.424	98.53%
PC21	0.54%	0.367	99.06%
PC22	0.40%	0.316	99.46%
PC23	0.34%	0.293	99.81%
PC24	0.17%	0.203	99.97%
PC25	0.03%	0.0823	100.00%

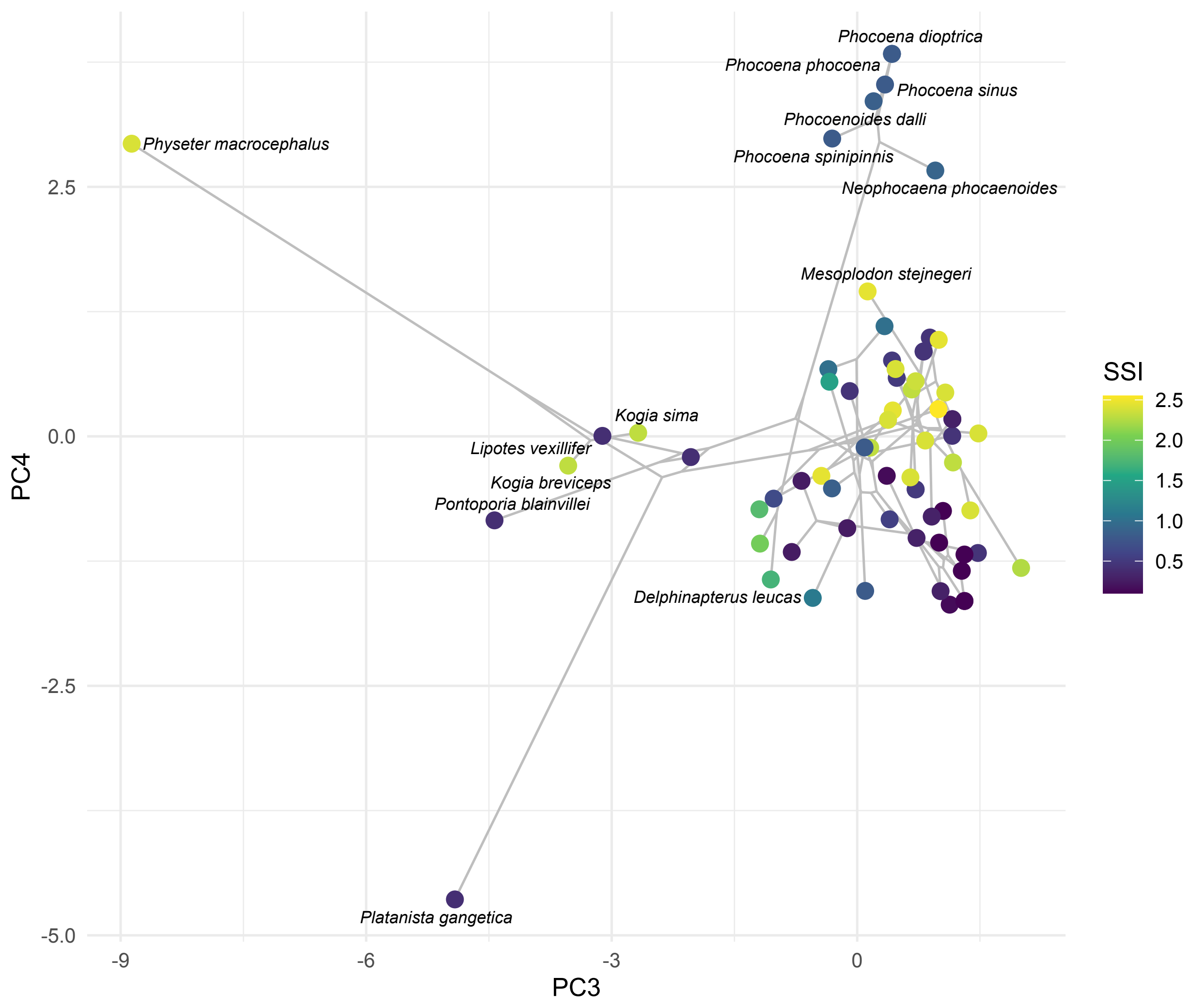

FIGURE S1

Resulting morphospace of the PCA on skull characters, with the suction specialization index (SSI) superimposed. PC3 (12.08% of variation) and PC4 (8.63% of variation) are plotted on the x- and y-axes, representing 20.71% of the total variation in the data. Color indicates SSI. Phylogeny was projected using a recent tree by Lloyd and Slater (2021).

List of Cranial and Mandibular Characters

Phylogenetic characters from Viglino et al. (2021) (via Meekin et al., 2024):

Rostrum

1. Length of rostrum as percent skull length: moderately long, 50–55% (0); long, 55–60% (1); very long, >60% (2); medium, 50–40% (3); very short, 40–35% (4).
2. Maxilla length as percent rostrum length: short, 89%, tips of maxillae to within 10% of rostrum tip (1); same as state 1 except lack of alveoli (2).
3. Lateral margin of rostrum anterior to maxillary flange: concave (0); straight (1); convex (2); absent (3).
4. Width of rostrum at antorbital notch as percent greatest width of maxillae at level of postorbital processes: wide >68% (0); medium, 68-45% (1); narrow, <45% (2).

Mandible

5. Length of mandibular symphysis as percent of mandible length: long, >20% (0); short, 69% of that distance (3).
6. Elevation of coronoid process: very high (0); moderate (1); low (2).

Orbit

7. Antorbital process of maxilla in dorsal view: triangular (0); robust and globose or rectilinear (1); absent (2).
8. Shape of postorbital process of frontal: robust, blunt descending posteriorly (0); pointed, attenuated, or acute triangular (1); triangular, trapezoidal, or an anteroposteriorly widened falciform (2); dorsoventrally long falciform (3).

Facial Region

9. Pneumatic maxillary crest overhanging medially: absent (0); present (1).
10. Premaxillae in dorsal view: separated anterior to bony nares, exposing mesethmoid (0); joined premaxillae (or maxillae) closing at least posterior part of mesorostral groove (1).
11. Ratio of width of right premaxilla to width of left premaxilla in line with midpoint of external nares: 0.90–1.19 (0); 1.20–1.50 (1); 1.50> (2).
12. Ratio of greatest width of premaxillae to greatest width of maxillae at level of postorbital processes: ≥0.50 (0); 0.49–0.38 (1); 70% (2).
13. Supracranial basin: absent (0); present (1).

Vertex and Area Adjacent to the Nares

14. Vertex: absent (0); present (1); highly developed (2).
15. Cranial vertex skewed asymmetrically to left side: absent (0); present (1).
16. Nuchal crest: higher than frontals and/or nasals (0); at same level as frontals and/or nasals (1); below frontals and/or nasals (2).

Temporal Fossae, Zygomatic Arch, and Occipitals

17. Parietals in dorsal view: contacting each other on the midline or separated by interparietal (0); in skull roof but visible only as small triangular areas at edges of intertemporal constriction, with supraoccipital overlapping and obscuring median portions (1); completely absent in skull roof (2); visible only as triangular areas, dorsolateral to supraoccipital, with non-overlapping supraoccipital separated from and contacting parietals along irregular suture (3).
18. Sagittal crest for temporal muscle: present (0); absent (1).
19. Zygomatic process of squamosal: directed anterolaterally (0); directed anteriorly (1).
20. Postglenoid process of squamosal: not reduced (0); greatly reduced (1).

Anterior Basicranium

21. Palatine exposure: exposed ventrally (0); partially covered by pterygoid, which divides it into medial and lateral exposures (1); ventral surfaces completely covered by pterygoids (2).

Posterior Basicranium

22. Length of zygomatic process of squamosal as percent of greatest width of maxilla at postorbital process: >31% (0); ≤30% (1).
23. Angle formed by basioccipital crests in ventral view: parallel with no angle formed (0); ca. 15–40° (1); ca. 42–68° (2); ca. 70–90° (3); >100° (4).
24. Paroccipital process, skull in ventral view: posterior edge located well anterior to the posterior edge of condyle (0); posterior edge in transverse line with posterior edge of condyle (1).

Phylogenetic characters from Paolucci et al. (2025) :

25. Occipital shield (ordered): (0) convex and forming an angle of about 40° with the longitudinal axis of the rostrum; (1) as state 0 with an angle of about 60°; (2) flat or concave forming an angle of about 90°; (3) flat or concave forming an angle distinctly greater than 90°.
